# Evaluation of lipid oxidation mechanisms in beverages and cosmetics via analysis of lipid hydroperoxide isomers

**DOI:** 10.1038/s41598-019-43645-1

**Published:** 2019-05-14

**Authors:** Junya Ito, Marina Komuro, Isabella Supardi Parida, Naoki Shimizu, Shunji Kato, Yasuhiro Meguro, Yusuke Ogura, Shigefumi Kuwahara, Teruo Miyazawa, Kiyotaka Nakagawa

**Affiliations:** 1 Food and Biodynamic Chemistry Laboratory, Graduate School of Agricultural Science, Tohoku University, Sendai, Miyagi 980-8572 Japan; 2 Department of Cell Biology, Division of Host Defense Mechanism, Tokai University School of Medicine, Isehara, Kanagawa 259-1193 Japan; 3 Laboratory of Applied Bioorganic Chemistry, Graduate School of Agricultural Science, Tohoku University, Sendai, Miyagi 980-8572 Japan; 4 Food and Health Science Research Unit, Graduate School of Agricultural Science, Tohoku University, Sendai, Miyagi 980-8572 Japan; 5 Food and Biotechnology Innovation Project, New Industry Creation Hatchery Center (NICHe), Tohoku University, Sendai, Miyagi 980-8579 Japan

**Keywords:** Nutrition, Risk factors

## Abstract

Understanding of lipid oxidation mechanisms (e.g., auto-oxidation and photo-oxidation) in foods and cosmetics is deemed essential to maintain the quality of such products. In this study, the oxidation mechanisms in foods and cosmetics were evaluated through analysis of linoleic acid hydroperoxide (LAOOH) and linoleic acid ethyl ester hydroperoxide (ELAOOH) isomers. Based on our previous method for analysis of LAOOH isomers, in this study, we developed a new HPLC-MS/MS method that enables analysis of ELAOOH isomers. The HPLC-MS/MS methods to analyze LAOOH and ELOOH isomers were applied to food (liquor) and cosmetic (skin cream) samples. As a result, LAOOH and ELAOOH isomers specific to photo-oxidation, and ELAOOH isomers characteristic to auto-oxidation were detected in some marketed liquor samples, suggesting that lipid oxidation of marketed liquor proceeds by both photo- and auto-oxidation during the manufacturing process and/or sales. In contrast, because only LAOOH and ELAOOH isomers specific to auto-oxidation were detected in skin cream stored under dark at different temperatures (−5 °C–40 °C) for different periods (2–15 months), auto-oxidation was considered to be the major oxidation mechanism in such samples. Therefore, our HPLC-MS/MS methods appear to be powerful tools to elucidate lipid oxidation mechanisms in food and cosmetic products.

## Introduction

Lipid oxidation is one of the main problems of deterioration in food (e.g., edible oils and beverages) and cosmetic (e.g., skin cream) products, affecting their chemical, physical, and sensory properties^1–5^. The understanding of mechanisms of lipid oxidation in food or cosmetic products is deemed essential as we can construct effective prevention measures against the lipid oxidation in these commodities by identifying and targeting the responsible oxidation mechanisms. While peroxide value and acid value have become the most common indicators for lipid oxidation levels^6–9^, these analysis methods do not provide enough information about the mechanisms of lipid oxidation. In general, the mechanisms of lipid oxidation have been well known including photo-oxidation (singlet-oxygen induced oxidation) and auto-oxidation (radical oxidation), and it has been found that each mechanism forms different characteristic lipid hydroperoxides (LOOH) with different regio and geometric (*cis/trans*) isomers^10–12^. Based on this knowledge, some studies, including our studies, have proposed that the oxidation mechanisms of lipids can be deduced according to its characteristic isomers of LOOH^13–18^.

**Figure 1:**
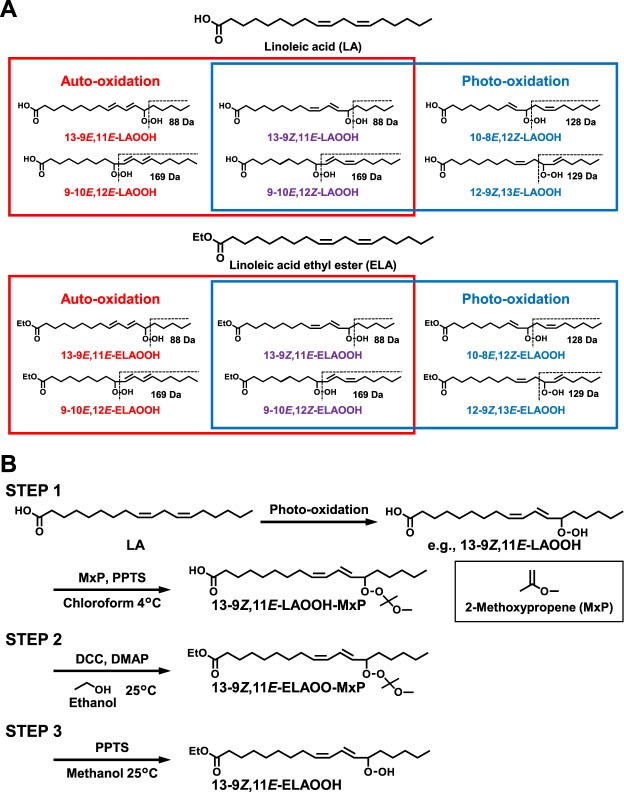
Chemical structures of six isomers of LA (9-10*E*, 12*Z*-LAOOH, 9-10*E*, 12*E*-LAOOH, 10-8*E*, 12*Z*-LAOOH, 12-9*Z*, 13*E*-LAOOH, 13-9*Z*, 11*E*-LAOOH, and 13-9*E*, 11*E*-LAOOH), ELA, and six isomers of ELAOOH (9-10*E*, 12*Z*-ELAOOH, 9-10*E*, 12*E*-ELAOOH, 10-8*E*, 12*Z*-ELAOOH, 12-9*Z*, 13*E*-ELAOOH, 13-9*Z*, 11*E*-ELAOOH, and 13-9*E*, 11*E*-ELAOOH). Depending on the type of oxidation (photo-oxidation or auto-oxidation), different LAOOH or ELAOOH isomers are formed (**A**). The preparation scheme of ELAOOH isomers (**B**).

While the lipid composition in food and cosmetic products vary depending on its source and production method, linoleic acid (LA) and linoleic acid ethyl ester (ELA) were found to be the major fatty acid constituent in food and cosmetic products^17,19–21^. Photo-oxidation of LA (or ELA) resulted in formation of 9-10*E*, 12*Z*-hydroperoxyl octadecadienoic acid (9-10*E*, 12*Z*-LAOOH), 10-8*E*, 12*Z*-LAOOH, 12-9*Z*, 13*E*-LAOOH, and 13-9*Z*, 11*E*-LAOOH (or 9-10*E*, 12*Z*-hydroperoxyl octadecadienoic acid ethyl ester (9-10*E*, 12*Z*-ELAOOH), 10-8*E*, 12*Z*-ELAOOH, 12-9*Z*, 13*E*-ELAOOH, and 13-9*Z*, 11*E*-ELAOOH)^16,22^, whereas auto-oxidation yielded 9-10*E*, 12*E*-LAOOH, 9-10*E*, 12*Z*-LAOOH, 13-9*E*, 11*E*-LAOOH, and 13-9*Z*, 11*E*-LAOOH (or 9-10*E*, 12*E*-ELAOOH, 9-10*E*, 12*Z*-ELAOOH, 13-9*E*, 11*E*-ELAOOH, and 13-9*Z*, 11*E*-ELAOOH)^12,23^ (Fig. 1A). Thus, some researches including ours were largely focusing on the identification of known LA or ELA hydroperoxide isomers (i.e., LAOOH or ELAOOH) as the basis to determine the type of oxidation mechanisms that are most likely to be responsible for food and cosmetics deterioration. To achieve this, an efficient yet reliable and highly-accurate analytical method was a necessity and therefore, we developed a method to analyze the regio and *cis/trans* isomers of LAOOH using HPLC-MS/MS in the presence of sodium ions^16,24^. While we were able to obtain a comprehensive profile of the LAOOH isomers in edible oils using this method^16^, analysis of food products with more complex composition has not been carried out. Moreover, ethyl esters of fatty acids (e.g., ELA) are considered to be important for the flavor of food. Since oxidized fatty acids and ethyl esters of fatty acids affect various odors, it is very important to clarify the content and formation mechanisms of LAOOH and ELAOOH, which are the primary oxidation products of fatty acids (LA) and ethyl esters of fatty acids (ELA). Moreover, ethyl esters of fatty acids (e.g., ELA) are considered to be important for the flavor of food. Since oxidized fatty acids and ethyl esters of fatty acids affect various odors, it is very important to clarify the content and formation mechanisms of LAOOH and ELAOOH, which are the primary oxidation products of fatty acids (LA) and ethyl esters of fatty acids (ELA). On top of that, because there is no analytical method to analyze ELAOOH, the measurement of ELAOOH isomers in food and cosmetic products were not conducted. In order to determine the responsible mechanisms related to the oxidative changes, it would be vital to investigate the composition of LAOOH and ELAOOH isomers that were present in foods and cosmetics.

Therefore, in this study, we developed an analytical method to analyze the regio and geometric isomers of ELAOOH based on our previously established HPLC-MS/MS analytical method for LAOOH. We then implemented these analytical methods (for LAOOH isomers and ELAOOH isomers) to obtain a comprehensive analysis of commodities samples (i.e., beverage (liquor) and cosmetic (skin cream)) which then will be used as the basis to determine the significant deteriorative lipid oxidation mechanisms in both products. The developed HPLC-MS/MS method appears to be a powerful tool to elucidate lipid oxidation mechanisms in food and cosmetic products.

## Materials and Methods

### Materials

LA, pyridinium *p*-toluenesulfonate (PPTS), and chloroform-d (CDCl_3_) were obtained from Sigma (St. Louis, MO, USA). Methylene blue, 2-methoxypropene (MxP), N,N’-dicyclohexylcarbodiimide (DCC), 4-(dimethylamino)pyridine (DMAP), and dimethyl sulfone were purchased from Wako Pure Chemical Co. (Osaka, Japan). All other reagents were of the highest grade available.

### Preparation of the LAOOH and ELAOOH isomers

Six isomers of ELAOOH were synthesized in three steps from a mixture of LAOOH isomers prepared by photo-oxidation of LA. (Fig. 1B): STEP 1, LAOOH isomers were prepared and treated with MxP (LAOO-MxP) to protect the hydroperoxyl group^10,25^; STEP 2, LAOO-MxP was esterified with ethanol to form ELAOO-MxP; STEP 3, the synthesized ELAOO-MxP was deprotected, and the resultant ELAOOH isomers were purified. Detailed conditions of steps 1–3 are as follows:

**STEP 1**: Six isomers of LAOOH (i.e., 9-10*E*, 12*Z*-LAOOH, 9-10*E*, 12*E*-LAOOH, 10-8*E*, 12*Z*-LAOOH, 12-9*Z*, 13*E*-LAOOH, 13-9*Z*, 11*E*-LAOOH, and 13-9*E*, 11*E*-LAOOH) were prepared from LA according to the previous method with slight modifications^16^. In brief, LA (5 g) was dissolved in 1 L of chloroform containing 20 μM methylene blue, then placed under an 18 W LED light (CN-304; Guangdong Nanguang Photo&Video Systems Co., Guangdong, China) for 48 h at 15 °C. The lamp was held 10 cm vertically above the sample, and the illumination intensity was set to approximately 50,000 lux for the first 24 h and 15,000 lux for another 24 h. To remove the methylene blue, the resultant sample was subsequently passed through a Sep-Pak silica column (500 mg, Waters, Milford, MA, USA) previously equilibrated with 12 mL of chloroform. The eluate was evaporated, and the resultant residue was dissolved in 4 mL of chloroform. A portion of the solution in chloroform (10 µL) was dried up, then the resultant residue was re-dissolved in hexane. The hexane solution was subjected to HPLC-UV (Condition 1, Table 1) to confirm the six LAOOH isomers. To isolate the LAOOH isomers, the sample in chloroform was subjected to semipreparative HPLC-UV (Condition 2, Table 1). Each isomer was isolated, then individually subjected twice to semipreparative HPLC-UV under the same conditions. Because 9-10*E*, 12*Z*-LAOOH and 9-10*E*, 12*E*-LAOOH were not entirely separated by silica columns, these isomers were subjected to semipreparative HPLC-UV (Condition 3, Table 1). To remove the acetic acid from mobile phase of semipreparative HPLC-UV (Condition 3, Table 1), the collected fractions were evaporated, then the residue was reconstituted with 30% methanol solution. The solution was loaded on to a SepPak Vac C18 column (5 g, Waters) previously equilibrated with methanol–water mixture (30:70, v/v). Following the sample loading, the column was washed with methanol–water (30:70, v/v) and the LAOOH were eluted with methanol. The eluent was collected, evaporated, and the residuere was dissolved in hexane. These solutions were subjected to HPLC-UV (Condition 1, Table 1) to confirm the purity of each isomer.

**Table 1:** HPLC conditions of HPLC-UV, HPLC-MS/MS and semipreparative HPLC-UV.

Parameters	Condition 1	Condition 2	Condition 3	Condition 4	Condition 5	Condition 6	Condition 7	Condition 8	Condition 9	Condition 10
Mobile phase	Mp 1	Mp 1	Mp 2	Mp 3	Mp 4	Mp 4	Mp 5	Mp 5	Mp 6	Mp 7
Flow rate (mL/min)	2.0	18.0	20.0	1.0	1.0	20.0	20.0	2.0	Gradient 1	Gradient 2
Column	Column 1	Column 2	Column 3	Column 4	Column 4	Column 3	Column 2	Column 1	Column 5	Column 6
Oven (°C)	40	40	40	40	40	40	40	40	40	40

Mp 1: hexane–2-propanol–acetic acid (100:1:0.1, v/v/v). Mp 2: methanol–water–acetic acid (100:30:0.1, v/v/v). Mp 3: methanol–water–acetic acid (100:20:0.1, v/v/v). Mp 4: methanol–water (100:15, v/v). Mp 5: hexane–2-propanol (100:0.3, v/v). Mp 6: (A); water–acetic acid (99.9: 0.1, v/v), (B); methanol–acetic acid (99.9: 0.1, v/v), 0–5.0 min, 60% B; 5.0–15.0 min, 60–100% B; 15.0–20.0 min, 100% B. Mp 7: (A); water, (B); methanol, 0–5.0 min, 80% B; 5.0–10.0 min, 80–90% B; 10.0–12.1 min, 90–100% B; 12.1–22.0 min, 100% B. Gradient 1: 0–5.0 min, 0.3 mL/min; 5.0–20.0 min, 0.2 mL/min, 20.0–25.0 min, 0.3 mL/min. Gradient 2: 0–5.0 min, 0.3 mL/min; 5.0–22.0 min, 0.2 mL/min, 22.0–27.0 min, 0.3 mL/min. Column 1: Inertsil SIL-100A (5 µm, 4.6 × 250 mm, GL science, Tokyo, Japan). Column 2: Inertsil SIL (5 µm, 10 × 250 mm, GL Sciences) connected with Unison UK-Silica (3 µm, 10 × 250 mm, Imtakt Corp., Kyoto, Japan). Column 3: Inertsil ODS-3 (5 µm, 10 mm × 250 mm, GL Sciences). Column 4: COSMOSIL 5C18-MS-II (5 µm, 4.6 × 250 mm, Nacalai tesque, Kyoto, Japan). Column 5: Inertsil ODS-3 (5 µm, 2.1 mm × 150 mm, GL Sciences). Column 6: COSMOSIL 5C18-MS-II (5 µm, 2.1 × 150 mm, Nacalai tesque). In Condition 9 and 10, the column eluent was mixed with methanol containing 2 mM sodium acetate. The flow rate of the methanol containing 2 mM sodium acetate was set to 0.01 mL/min.

To protect the hydroperoxyl group of LAOOH (i.e., synthesis of LAOO-MxP isomers), MxP (67 µL, 1/20 vol. eq. of sample solution) and a solution of PPTS (67 µL, 40 mM in chloroform) were added to the solution of each isomer of LAOOH (20 mg) in chloroform (1.3 mL) and the reaction mixture was stirred for 10 min at 4 °C. Then, each sample solution was loaded on to a Strata Silica column (500 mg, Phenomenex, Torrance, CA, USA) and eluted with chloroform. These solutions then were evaporated, and the residue was dissolved in chloroform. A portion of the chloroform solution (10 µL) was dried up and the residue was re-dissolved in methanol. The methanol samples were subjected to HPLC-UV (Condition 4, Table 1) to confirm the LAOO-MxP.

**STEP 2**: To obtain ELAOO-MxP, a solution of DCC (12.9 mg) and DMAP (6.4 mg) in dehydrated ethanol (2.64 mL) was added into 20 mg of each LAOO-MxP isomers. The mixture was incubated for 12 h at 25 °C and subjected to HPLC-UV (Condition 5, Table 1) to confirm the generation of esterified LAOO-MxP (i.e., ELAOO-MxP). Once confirmed, each reacted mixture was evaporated, dissolved in 3 mL of hexane–diethyl ether mixture (2:1, v/v), and then passed through a Strata silica cartridge (500 mg, Shimadzu) previously equilibrated with 12 mL of hexane–diethyl ether mixture (2:1, v/v). Finally, the eluate was evaporated, then the residue was re-dissolved in 1 mL of methanol. The methanol solution was subjected to semipreparative HPLC-UV (Condition 6, Table 1) to obtain pure ELAOO-MxP isomers.

**STEP 3**: The fractions containing ELAOO-MxP were evaporated and the residue was dissolved in methanol (50 mg/mL), into which a solutioin of PPTS in methanol (100 mM, 1/1 vol. eq.) was added. The solutions were kept at 25 °C for 1 hour. To the reaction mixture were added water for yielding a mixed solution of methanol–water (30:70, v/v). The mixture was then loaded on to a Strata C18 column (500 mg, Phenomenex) previously equilibrated with 12 mL of methanol–water mixture (30:70, v/v). Following the sample loading, the column was washed with methanol–water (30:70, v/v) and the ELOOH were eluted with methanol. The eluent was collected, evaporated, and the residue was re-dissolved in chloroform. A portion of the sample was then subjected to semipreparative HPLC-UV (Condition 7, Table 1) to obtain six ELAOOH isomers. The purity of each isomers was confirmed using HPLC-UV (Condition 8, Table 1). The geometric chemical structure (*E-Z* configuration) of LAOOH and ELAOOH isomers were confirmed by ^1^H NMR using a Varian Unity Plus-400 spectrometer (Varian, Palo Alto, CA, USA) at 400 MHz with CDCl_3_ as the solvent. In order to determine the concentration of LAOOH and ELAOOH, each LAOOH and ELAOOH isomer was subjected to quantitative ^1^H NMR with dimethyl sulfone as a standard substance. The signal assigned to the proton of the hydroperoxyl group was compared with those of the methyl group of dimethyl sulfone. Each prepared isomer was used as a reference standard for quantitative analysis of LAOOH and ELOOH in liquor and cosmetics as described in the following sections.

### MS/MS and HPLC-MS/MS analysis of LAOOH and ELAOOH isomers

A portion of each solution of LAOOH and ELAOOH isomer was evaporated and the residue was dissolved in methanol containing sodium acetate (0.1 mM). For analysis of fragment ions, each sample was directly infused to the microOTOF-Q II mass spectrometer (Bruker Daltonik, Bremen, Germany) at flow rate of 150 µL/h. The analytical conditions of Q1 and MS/MS analysis used in the current study are shown in Table 2.

**Table 2:** Analytical conditions used for Q1 and MS/MS analysis.

Parameters	LAOOH	ELAOOH
	Q1 analysis	
Source	ESI	ESI
Ion polarity	Positive	Positive
Mass range (*m/z*)	20–400	20–400
End plate offset (V)	500	500
Capillary (V)	4000	4000
Nebulizer (Bar)	0.4	0.4
Dry gas (L/min)	4.0	4.0
Dry temp (°C)	180.0	180.0
Funnel 1 RF (Vpp)	200.0	200.0
Funnel 2 RF (Vpp)	250.0	200.0
icCID energy (eV)	50.0	0.0
Hexapole RF (Vpp)	250.0	400.0
Ion energy (eV)	5.0	4.0
Low mass (*m/z*)	50.00	300.00
Collision energy (eV)	5.0	10.0
Collision RF (Vpp)	300.0	200.0
Transfer time (µs)	30.0	35.0
Pre pulse storage (µs)	7.0	10.0
	MS/MS analysis	
Mass	335.20	363.25
Width	10.0	5.0
isCID	0.0	0.0
Collision	10.0	25.0
× Acq.	1.0	1.0

ESI, electrospray ionization; RF, radio frequency; isCID, in-source collision induced dissociation.

The HPLC-MS/MS analysis system consisted of a liquid chromatography system (Shimadzu, Kyoto, Japan) equipped with vacuum degasser, a quaternary pump, and an autosampler, which coupled with a 4000 QTRAP quadrupole/linear ion trap tandem mass spectrometer (SCIEX, Tokyo, Japan). Using the identified fragment ions, multiple reaction monitoring (MRM) of each LAOOH and ELAOOH isomer was performed with the HPLC-MS/MS system (Table 3). A mixture of six LAOOH isomers (500 nM each isomer in methanol, 10 µL) and a mixture of six ELAOOH isomers (100 nM each isomer in methanol, 10 µL) were analyzed under Conditions 9 and 10 (Table 1), respectively.

**Table 3:** Analytical conditions used for MS/MS MRM analysis.

Compound	Precursor ion (*m*/*z*)	Product ion (*m*/*z*)	Source	Ion polarity	DP (V)	EP (V)	CE (V)	CXP (V)	Curtain gas (psi)	Ion spray voltage (V)	Temperature (°C)	Ion source gas 1 (psi)	Ion source gas 2 (psi)	Collision Gas (psi)
9-10*E*, 12*E*-LAOOH	335.2	195.1	ESI	Positive	46	10	14.8	10	20.0	5500.0	600.0	40.0	40.0	4.0
9-10*E*, 12*Z*-LAOOH	335.2	195.1	ESI	Positive	46	10	14.8	10	20.0	5500.0	600.0	40.0	40.0	4.0
10-8*E*, 12*Z*-LAOOH	335.2	207.1	ESI	Positive	51	10	20.7	10	20.0	5500.0	600.0	40.0	40.0	4.0
12-9*Z*, 13*E*-LAOOH	335.2	206.1	ESI	Positive	61	10	18.9	10	20.0	5500.0	600.0	40.0	40.0	4.0
13-9*E*, 11*E*-LAOOH	335.2	247.1	ESI	Positive	61	10	17.0	16	20.0	5500.0	600.0	40.0	40.0	4.0
13-9*Z*, 11*E*-LAOOH	335.2	247.1	ESI	Positive	61	10	17.0	16	20.0	5500.0	600.0	40.0	40.0	4.0
9-10*E*, 12*E*-ELAOOH	363.3	223.2	ESI	Positive	67	10	16.0	10	20.0	5500.0	600.0	40.0	60.0	4.0
9-10*E*,1 2*Z*-ELAOOH	363.3	223.2	ESI	Positive	67	10	16.0	10	20.0	5500.0	600.0	40.0	60.0	4.0
10-8*E*, 12*Z*-ELAOOH	363.3	235.3	ESI	Positive	67	10	21.5	12	20.0	5500.0	600.0	40.0	60.0	4.0
12-9*Z*, 13*E*-ELAOOH	363.3	234.2	ESI	Positive	67	10	19.5	12	20.0	5500.0	600.0	40.0	60.0	4.0
13-9*E*, 11*E*-ELAOOH	363.3	275.4	ESI	Positive	67	10	21.0	15	20.0	5500.0	600.0	40.0	60.0	4.0
13-9*Z*, 11*E*-ELAOOH	363.3	275.4	ESI	Positive	67	10	21.0	15	20.0	5500.0	600.0	40.0	60.0	4.0

ESI, electrospray ionization; DP, declustering potential; EP, entrance potential; CE, collision energy; CXP, Collision cell exit potential.

### Extraction and analysis of LAOOH and ELAOOH from liquors

Five different marketed liquor samples (four whiskies and one brandy) commercially sold in clear glass bottles, were immediately subjected to quantitative analysis once opened. Prior to that, 50 mL of each sample was mixed with water and prepared to 20% ethanol solution, then the solution was loaded on to a Strata C18 column (100 mg, Shimadzu) previously equilibrated with methanol–water mixture (30:70, v/v). The column was then washed with methanol–water (30:70, v/v) followed with elution of LAOOH and ELAOOH with methanol. The collected eluent was evaporated, and the residues were dissolved in 1 mL of methanol. Each sample solution (10 µL) was injected into the HPLC-MS/MS system for quantitative analysis of LAOOH and ELAOOH under Conditions 9 and 10 (Table 1), respectively. MS/MS conditions were shown in Table 3.

### Extraction and analysis of LAOOH and ELAOOH from cosmetics

Cosmetic samples (skin cream) that were previously stored under dark at different temperatures (−5 °C, 25 °C, and 40 °C) for different periods (2, 4, 6, and 15 months) were used in this analysis. Each sample (80 mg) was added with 4 mL of methanol and homogenized using vortex and sonicator. Next, 1 mL of the solution in methanol was centrifuged at 13,000 × g for 1 min at 25 °C, and the supernatants were collected. The collected supernatants (10 µL each) were injected into the HPLC-MS/MS system for quantitative analysis of LAOOH and ELAOOH under Conditions 9 and 10 (Table 1), respectively. MS/MS conditions were shown in Table 3.

## Results and Discussion

### Preparation of standard LAOOH and ELAOOH isomers for MS/MS analysis

**Figure 2:**
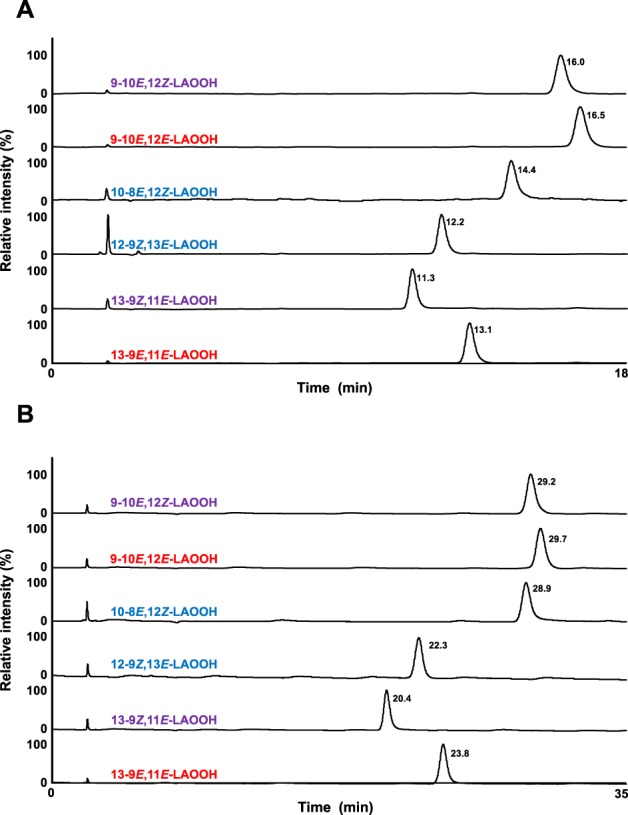
The UV chromatograms of LAOOH (**A**) and ELAOOH (**B**) isomers. The six isomers of LAOOH (50 µg/mL each) and ELAOOH (50 µg/mL each) were subjected to HPLC-UV with silica columns. Detailed analytical conditions are described in the Materials and Methods section.

Standard LAOOH and ELAOOH isomers, which are essential in the development of analytical methods for LAOOH and ELAOOH, were prepared through 3 steps (Fig. 1B). In agreement with many previous studies, photo-oxidation (singlet oxygen-induced oxidation) was employed to prepare six LAOOH isomers, and peaks corresponding to LAOOH isomers were detected as the oxidation products on the UV (210 nm) chromatogram (Supplementary Information 1). Then by performing semipreparative HPLC-UV with silica (Condition 2, Table 1), the six LAOOH isomers (i.e., 9-10*E*, 12*E*-LAOOH (57.4 mg), 9-10*E*, 12*Z*-LAOOH (142.3 mg), 10-8*E*, 12*Z*-LAOOH (31.9 mg), 12-9*Z*, 13*E*-LAOOH (42.7 mg), 13-9*E*, 11*E*-LAOOH (100.3 mg), and 13-9*Z*, 11*E*-LAOOH (181.0 mg)) were individually isolated. Because 9-10*E*, 12*Z*-LAOOH and 9-10*E*, 12*E*-LAOOH were not entirely separated by silica columns, these isomers were completely separated by semipreparative HPLC-UV with an ODS column (Condition 3, Table 1). Each isolated LAOOH isomer was subjected to HPLC-UV (Condition 1, Table 1), and detected as a single peak individually (Fig. 2A). The geometric isomerism (*E-Z* configuration) and purity were verified by ^1^H NMR (Supplementary Information 2A–F). From these results, we obtained pure LAOOH isomers that were used as standard reference for quantitative analysis of LAOOH isomers in beverage and cosmetic products.

Regarding the preparation of ELAOOH isomers, we initially attempted to isolate ELAOOH isomers from oxidized ELA using semipreparative HPLC-UV. However, unlike analysis of LAOOH isomers, it was very difficult to separate the six isomers of ELAOOH from each other by HPLC (data not shown). Therefore, we conceived the synthesis of ELAOOH by condensation reaction between carboxylic acid (i.e., LAOOH) and alcohol (i.e., ethanol). Since the hydroperoxyl group is unstable, we protected the hydroperoxyl group of LAOOH with MxP, yielding LAOO-MxP before the condensation reaction. Then, we successfully synthesized ELAOO-MxP by condensation reaction of LAOO-MxP and ethanol. Synthesized ELAOO-MxP was finally deprotected, and the resultant ELAOOH was chromatographically purified. Six ELAOOH isomers (i.e., 9-10*E*, 12*E*-ELAOOH (4.0 mg), 9-10*E*, 12*Z*-ELAOOH (9.3 mg), 10-8*E*, 12*Z*-ELAOOH (7.9 mg), 12-9*Z*, 13*E*-ELAOOH (8.6 mg), 13-9*E*, 11*E*-ELAOOH (5.5 mg), and 13-9*Z*, 11*E*-ELAOOH (7.8 mg)) were obtained and subjected to HPLC-UV (Condition 7, Table 1). Each isomer was detected as a single peak individually (Fig. 2B), inferring the high purity of the prepared ELAOOH isomers. The geometric isomerism (*E-Z* configuration) and purity were also verified by ^1^H NMR (Supplementary Information 3A–F). In the previous study, LAOO-MxP has been used for ester synthesis with glycerolipids (e.g., lysophosphatidylcholine and diacylglycerol)^10,11,17^, however it has been hardly applied to the condensation reaction with alcohol. Our study indicated that LOOH protected with MxP (e.g., LAOO-MxP) could also be applied for ester condensation with alcohols, and this technique is expected to be useful for preparing various LOOH standards.

### MS/MS analysis of LAOOH and ELAOOH isomers

In the previous study, we revealed that in the presence of sodium ions during MS/MS analysis, LAOOH isomers yielded structure-diagnostic fragment ions that were highly useful in identifying the position of the hydroperoxyl group^16,24^. For example, mass spectrum of sodiated 13-9*Z*, 11*E*-LAOOH (i.e., *m/z* 335.2 [M + Na]^+^) revealed a neutral loss of 88 Da arising from fragmentation of the hydroperoxyl group. Moreover, similar results were not only observed in other fatty acid hydroperoxide (i.e., arachidonic acid hydroperoxide) but also in phospholipid hydroperoxide, triacylglycerol hydroperoxide, and squalene hydroperoxide^17,26,27^. From these studies, we concluded that utilization of sodium ion during MS/MS analysis is highly useful for analysis of several LOOH including fatty acid ester hydroperoxide. Hence, in this study, we postulated that this method would also be useful for analysis of ELAOOH isomers. Accordingly, direct infusion of the six LAOOH and ELAOOH standards into the MS/MS system was performed in the presence of the sodium ion to investigate their fragmentations. Unless specifically stated otherwise, spectral data were obtained under the optimized conditions. Each spectrum is representative of at least a triplicate analysis.

**Figure 3:**
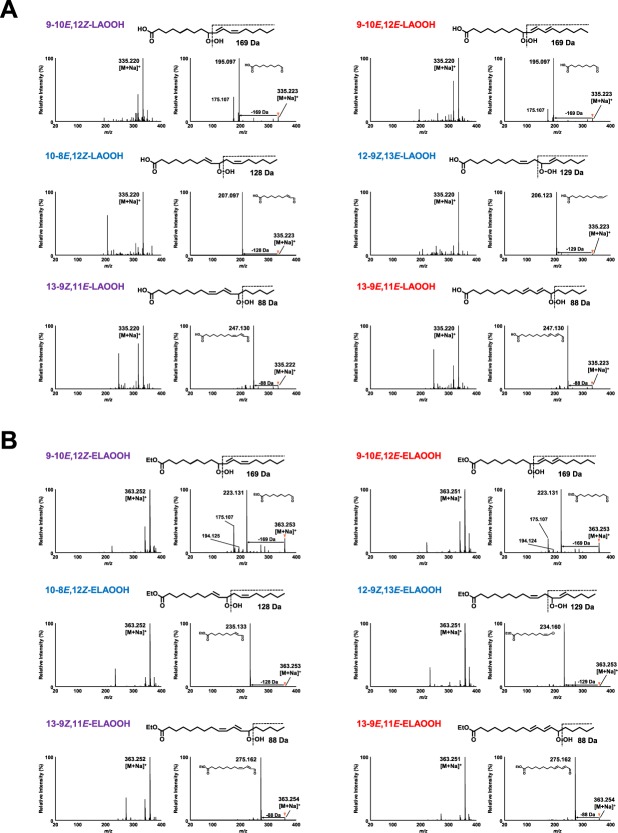
Q1 mass spectra and product ion mass spectra of LAOOH (**A**) and ELAOOH (**B**) isomers in the presence of the sodium ion (positive ion mode). LAOOH and ELAOOH isomers were dissolved in methanol containing 0.1 mM sodium acetate. The sample solutions (10 uM) were infused directly into a micrOTOF-Q II mass spectrometer at a flow rate of 150 µL/h. Detailed analytical conditions are described in the Materials and Methods section.

As similar to our previous study^24^, each sodiated molecular ion of LAOOH isomer generated the significant hydroperoxyl group-derived fragment ions (Fig. 3A). Using a similar method, ELAOOH isomers were also subjected to MS/MS analysis in the presence of the sodium ion, which yielded the same fragment patterns with LAOOH. For example, 13-9*Z*, 11*E*-ELAOOH produced a characteristic fragment ion at *m/z* 275.1 ([M + Na−C_5_H_12_O]^+^) corresponding to a 88 Da loss from the sodiated molecular ion (*m/z* 363.2 [M + Na]^+^) (Fig. 3B). As we expected, the fragmentation patterns of ELAOOH observed in this study were consistent with our previously reported analysis of LAOOH^16,24^ as well as other LOOH species in the presence of sodium^10,17,26^. Therefore, the ability of sodium ions to promote ionization of hydroperoxyl group derived fragment ions can be considered effective regardless of the type of LOOH.

### HPLC-MS/MS analysis of LAOOH and ELAOOH isomers

**Figure 4:**
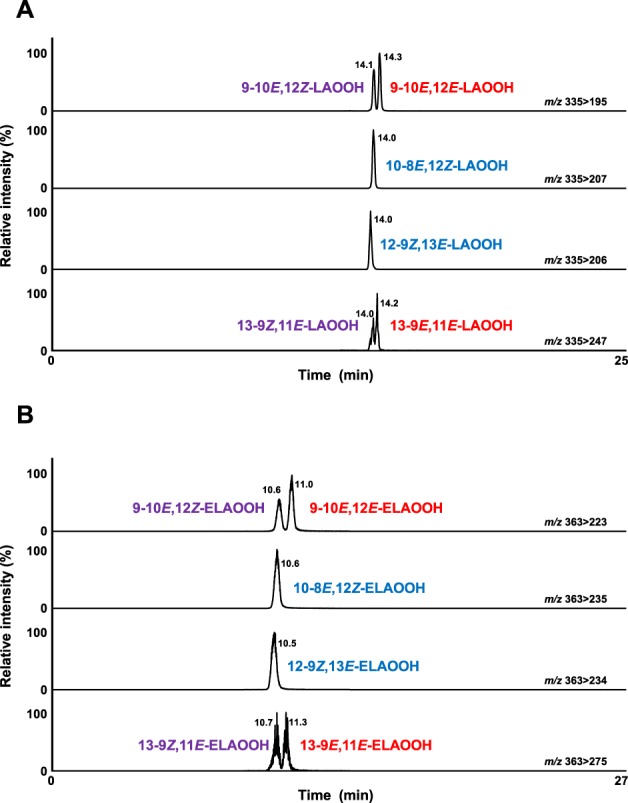
The MRM chromatograms of LAOOH (**A**) and ELAOOH (**B**) isomers. A mixture of six isomers of LAOOH (500 nM in methanol, 10 µL each) and a mixture of six isomers of ELAOOH (100 nM in methanol, 10 µL each) were analyzed by HPLC-MS/MS. Detailed analytical conditions are described in the Materials and Methods section.

HPLC-MS/MS method for LAOOH was developed according to the previous method^16^ with slight modifications (Condition 9, Table 1) (Table 3). In the MRM chromatograms, each LAOOH isomer was completely separated (Fig. 4A) and detected at the following retention times: 14.0 min (10-8*E*, 12*Z*-LAOOH), 14.0 min (12-9*Z*, 13*E*-LAOOH), 14.0 min (13-9*Z*, 11*E*-LAOOH), 14.1 min (9-10*E*, 12*Z*-LAOOH), 14.2 min (13-9*E*, 11*E*-LAOOH), and 14.3 min (9-10*E*, 12*E*-LAOOH). By modifying the previously reported method where detection limits of LAOOH isomers were pmol levels^16^, detection limits were enhanced to 25 fmol. Then, we also successfully discriminated six ELAOOH isomers using HPLC-MS/MS with an ODS column (Condition 10, Table 1) (Fig. 4B) and MRM analysis based on fragments observed during product ion scanning of each isomer in the presence of the sodium ion (Table 3). Each ELAOOH isomer was detected at the following retention times: 10.5 min (12-9*Z*, 13*E*-ELAOOH), 10.6 min (9-10*E*, 12*Z*-ELAOOH), 10.6 min (10-8*E*, 12*Z*-ELAOOH), 10.7 min (13-9*Z*, 11*E*-ELAOOH), 11.0 min (9-10*E*, 12*E*-ELAOOH), and 11.3 min (13-9*E*, 11*E*-ELAOOH). To the best of our knowledge, this is the first complete discrimination of ELAOOH isomers using HPLC-MS/MS analysis. The detection limit of each ELAOOH isomer was 0.1–2.0 fmol. These methods may therefore be useful in the quantification of LAOOH and ELAOOH isomers on commodities samples and the evaluation of lipid oxidation mechanisms.

### Evaluation of lipid oxidation mechanisms of food and cosmetic samples

**Figure 5:**
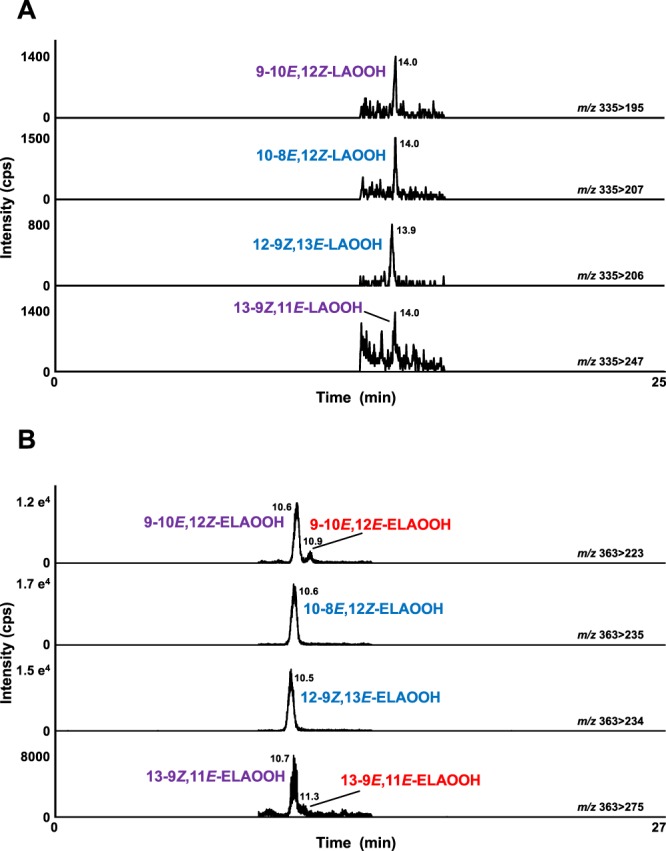
The MRM chromatograms of LAOOH (**A**) and ELAOOH (**B**) isomers in brandy sample. Extract from brandy sample (10 µL) was analyzed by HPLC-MS/MS. Detailed analytical conditions are described in the Materials and Methods section.

**Figure 6:**
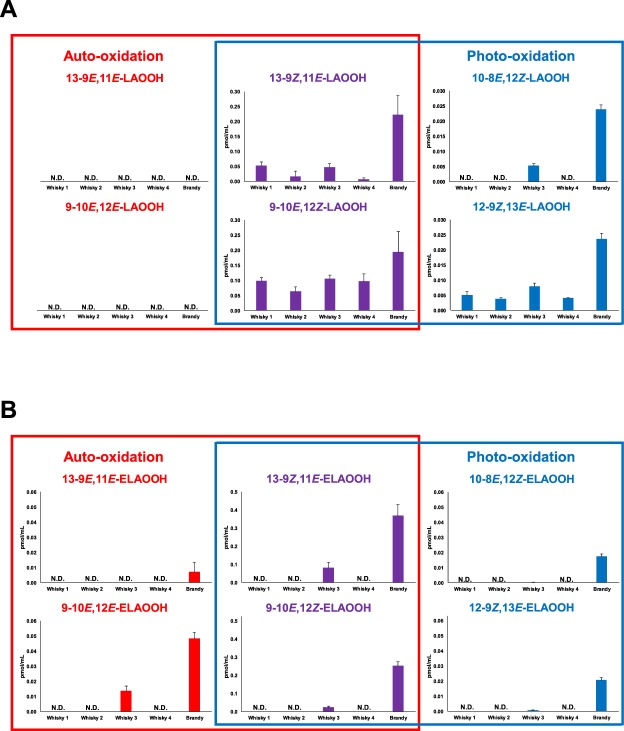
The concentrations of LAOOH (**A**) and ELAOOH (**B**) isomers of marketed liquor samples (Mean ± SD (n = 3)). Extract from liquor samples (four whiskies and one brandy) were analyzed by HPLC-MS/MS with MRM mode. Detailed analytical conditions are described in the Materials and Methods section.

Subsequently, we implemented analytical methods for LAOOH and ELAOOH isomers to obtain a comprehensive analysis of commodities. Some alcoholic beverages such as beer and whisky are known to contain fatty acids and fatty acid ethyl esters^19,28,29^. Lipid peroxidation of these fatty acids and fatty acid ethyl esters have been reported to affect the flavor of foods. Therefore, we focused on liquor as a food (beverage) sample in this study. We analyzed five different marketed liquor samples, including four whisky and one brandy. The typical chromatograms of liquor are shown in Fig. 5. LAOOH and ELAOOH isomers were detected in one whisky sample and brandy despite being analyzed immediately after opening (Figs 5 and 6). LAOOH isomers that were detected in liquors were the isomers characteristic of photo-oxidation (i.e., 9-10*E*, 12*Z*-LAOOH, 10-8*E*, 12*Z*-LAOOH, 12-9*Z*, 13*E*-LAOOH, and 13-9*Z*, 11*E*-LAOOH). On the other hand, the ELAOOH isomers formed by both photo-oxidation (i.e., 9-10*E*, 12*Z*-ELAOOH, 10-8*E*, 12*Z*-ELAOOH, 12-9*Z*, 13*E*-ELAOOH, and 13-9*Z*, 11*E*-ELAOOH) and auto-oxidation (i.e., 9-10*E*, 12*Z*-LAOOH, 9-10*E*, 12*E*-LAOOH, 13-9*Z*, 11*E*-LAOOH, and 13-9*E*, 11*E*-LAOOH) were detected in liquor samples (Fig. 6). The detection of auto-oxidation products of ELA but not LA in whisky 3 and brandy might have been due to the higher detection sensitivity of ELAOOH. The detection limits of ELAOOH isomers are about 25 times higher than LAOOH isomers; thus, ELAOOH isomers characteristic of auto-oxidation (i.e., 9-10*E*, 12*E*-ELAOOH and 13-9*E*, 11*E*-ELAOOH) were detected. Such results suggested that both photo- and auto-oxidation had already occurred in marketed liquor before opening (e.g., during the manufacturing process and/or sales). Also, the concentration of fatty acid and fatty acid ethyl ester in whisky is mainly affected by the manufacturing process^30^, and thus may explain the presence/absence of LAOOH and ELAOOH in whisky samples in this study. Among the measured liquor samples, brandy contained a relatively high concentration of ELAOOH isomers, and therefore the brandy analyzed in this study might have contained certain compounds that acted as a prooxidant. Considering that LAOOH and ELAOOH isomers characteristic of photo-oxidation were detected, photosensitizers that induce photo-oxidation might have been contained. Formation of LOOH contributes to the emergence of offensive odor and taste^17,31^, and so, preventing the generation of LOOH in liquor (i.e., suppression of photo-oxidation) could be essential for maintaining the quality of liquor.

**Figure 7:**
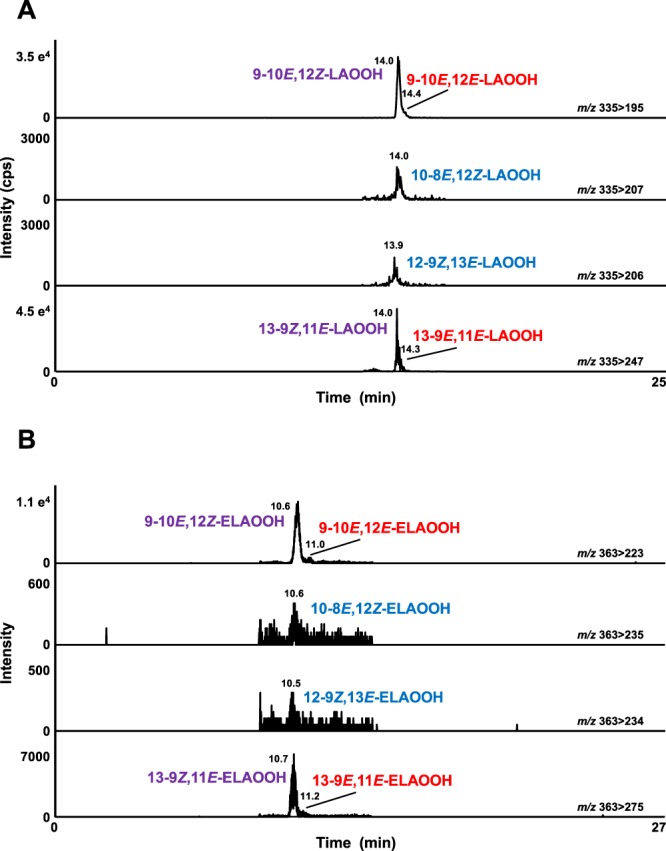
The MRM chromatograms of LAOOH (**A**) and ELAOOH (**B**) isomers in cosmetic samples. Extract from cosmetic sample (10 µL) was analyzed by HPLC-MS/MS. Detailed analytical conditions are described in the Materials and Methods section.

**Figure 8:**
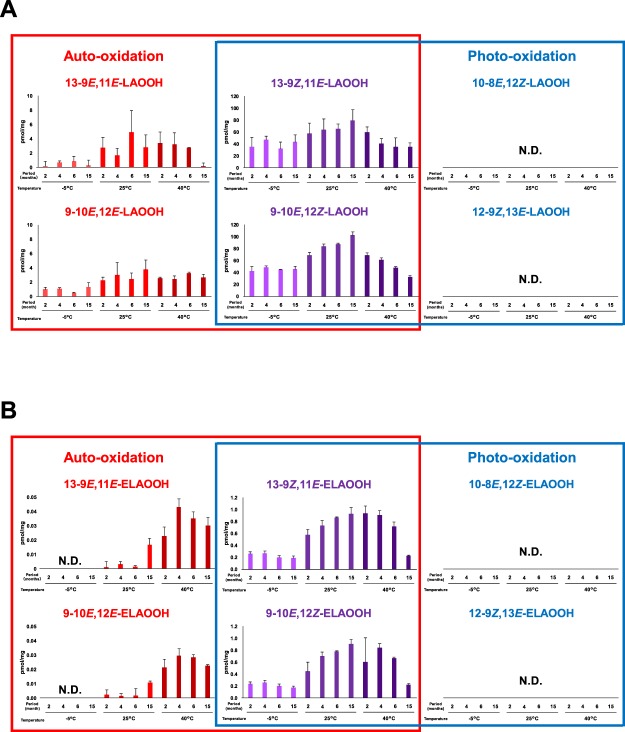
The concentrations of LAOOH (**A**) and ELAOOH (**B**) isomers of cosmetic samples (Mean ± SD (n = 3)). Cosmetic samples were stored under dark at different temperatures (−5 °C, 25 °C, 40 °C) for different periods (2, 4, 6, 15 months). Extract from cosmetic samples were analyzed by HPLC-MS/MS with MRM mode. Detailed analytical conditions are described in the Materials and Methods section.

In addition to liquor, cosmetics (e.g., skin cream) are also known to contain fatty acids and fatty acid ethyl esters^2,4,21^. Furthermore, the oxidation of lipids contained in cosmetics could lead to the deterioration of quality. Thomsen *et al*. reported that odor-causing substances (e.g., hexanal) are formed in cosmetics depending on storage temperature, light-exposure, and time^4^. However, the oxidation mechanisms of lipids in cosmetics have not been evaluated, furthermore, the influence of storage conditions on oxidation mechanisms have not been assessed. In this study, in order to evaluate the influence of the storage temperature and period on oxidation mechanisms, skin cream samples stored under dark at different temperatures (−5 °C, 25 °C, and 40 °C) for different period of times (2, 4, 6, and 15 months) were analyzed. LAOOH and ELAOOH isomers were then extracted and analyzed by the HPLC-MS/MS method. In contrast to liquor samples, auto-oxidized LAOOH isomers (i.e., 9-10*E*, 12*E*-LAOOH, 9-10*E*, 12*Z*-LAOOH, 13-9*E*, 11*E*-LAOOH, and 13-9*Z*, 11*E*-LAOOH) and ELAOOH isomers (i.e., 9-10*E*, 12*E*-ELAOOH, 9-10*E*, 12*Z*-ELAOOH, 13-9*E*, 11*E*-ELAOOH, and 13-9*Z*, 11*E*-ELAOOH) were mainly detected in skin cream (Figs 7 and 8). This result suggested that auto-oxidation occurred in skin cream under dark and confirmed the influence of storage temperature and term on lipid oxidation. When the skin cream was stored at −5 °C, each concentration of LAOOH isomers and ELAOOH isomers were not altered despite the difference in storage period (Fig. 8). On the other hand, the LAOOH isomers and ELAOOH isomers in skin cream stored at 25 °C were increased depending on the stored periods. As auto-oxidation of lipids tend to proceed at high temperature, it is most likely that low-temperature storage (i.e., −5 °C), inhibits the oxidation process. In contrast, the concentrations of LAOOH and ELAOOH isomers in skin cream stored at 40 °C tended to either decrease throughout the storage time or increase for a short time period and then later decrease during storage. This result seems to be due to the lipid oxidation being accelerated by high temperature and then LOOH being decomposed. In fact, the sample stored at 40 °C for 15 months exhibited significant change in color and smell. From these results, we found that it was possible to evaluate lipid oxidation mechanisms (i.e., auto-oxidation) in cosmetics with the use of the developed method, and to determine the possibly effective defense method (i.e., storage at low temperature) to suppress the progress of oxidation.

## Conclusions

In this study, LAOOH and ELAOOH isomers were prepared. Using MS/MS analysis in the presence of the sodium ion, like LAOOH, we were able to discriminate each ELAOOH isomer based on their distinct fragmentation patterns. Based on these findings, an analytical method using HPLC-MS/MS was developed to analyze the regio and geometric (*cis/trans*) isomers of ELAOOH. We tried to quantify the LAOOH and ELAOOH isomers content in liquor and cosmetic samples and determined the lipid oxidation mechanisms that are most likely to be responsible for the deterioration of each samples. As a result, both photo-and auto-oxidation were found to be the oxidation mechanism that occurred in marketed liquor samples prior to opening, especially in brandy. In contrast, auto-oxidation was considered to be the major oxidation mechanism responsible for deterioration of cosmetic samples during the storage under dark conditions, with its severity varied depending on the storage temperature and period. Overall, the recently developed HPLC-MS/MS method appears to be a powerful tool to elucidate lipid oxidation mechanisms in food and cosmetic products, and thus providing useful information that may serve as the basis to construct effective preventive strategy for lipid oxidation as part of products quality control and improvement.

## Supplementary information

Supplementary Information
