# Supplementary material for: Evaluation of lipid oxidation mechanisms in beverages and cosmetics via analysis of lipid hydroperoxide isomers
Source: Sci Rep. 2019 May 14;9:7387. doi: 10.1038/s41598-019-43645-1 (PMC6517444; doi:10.1038/s41598-019-43645-1)
Supplement: Supplementary file 1 — Supplementary Information [file 41598_2019_43645_MOESM1_ESM.pdf]

Supplementary Information

Evaluation of lipid oxidation mechanisms in beverages and cosmetics via analysis of lipid hydroperoxide isomers

Junya Ito[1], Marina Komuro[1], Isabella Supardi Parida[1], Naoki Shimizu[1], Shunji Kato[2], Yasuhiro Meguro[3], Yusuke Ogura[3], Shigefumi Kuwahara[3], Teruo Miyazawa[4,5], Kiyotaka Nakagawa[1,*]

[1]Food and Biodynamic Chemistry Laboratory, Graduate School of Agricultural Science, Tohoku University, Sendai, Miyagi, 980-8572, Japan

[2]Department of Cell Biology, Division of Host Defense Mechanism, Tokai University School of Medicine, Isehara, Kanagawa, 259-1193, Japan

[3]Laboratory of Applied Bioorganic Chemistry, Graduate School of Agricultural Science, Tohoku University, Sendai, Miyagi, 980-8572, Japan

[4]Food and Health Science Research Unit, Graduate School of Agricultural Science, Tohoku University, Sendai, Miyagi, 980-8572, Japan

[5]Food and Biotechnology Innovation Project, New Industry Creation Hatchery Center (NICHe), Tohoku University, Sendai, Miyagi, 980-8579, Japan

*Corresponding Author: nkgw@m.tohoku.ac.jp

Supplementary Information 1

The UV chromatogram of photo-oxidized LA. Photo-oxidized LA was subjected to HPLC-UV (210 nm) with silica columns to isolate the six LAOOH isomers (peaks 1-6). Detailed analytical conditions are described in the Materials and Methods section.

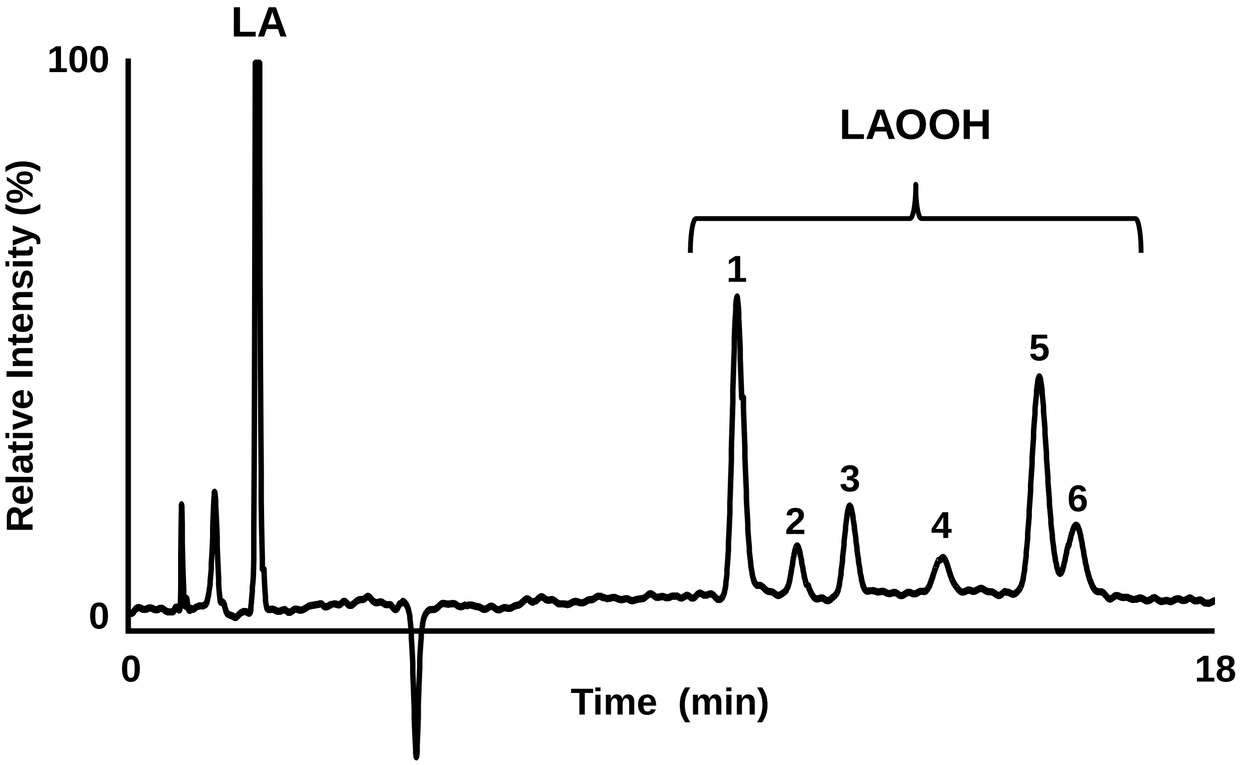

Supplementary Information 2

^{1}H NMR spectra of LAOOH isomers (9-10*E*,12*Z*-LAOOH (A), 9-10*E*,12*E*-LAOOH (B), 10-8*E*,12*Z*-LAOOH (C), 12-9*Z*,13*E*-LAOOH (D), 13-9*Z*,11*E*-LAOOH (E), 13-9*E*,11*E*-LAOOH (F)). Detailed analytical conditions are described in the Materials and Methods section.

Supplementary Information 2

A

^{1}H NMR (400 MHz, $CDCl_3$)

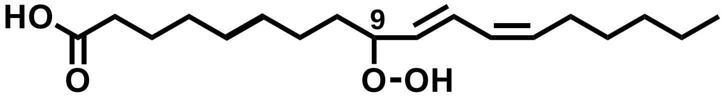

9-10*E*,12*Z*-LAOOH

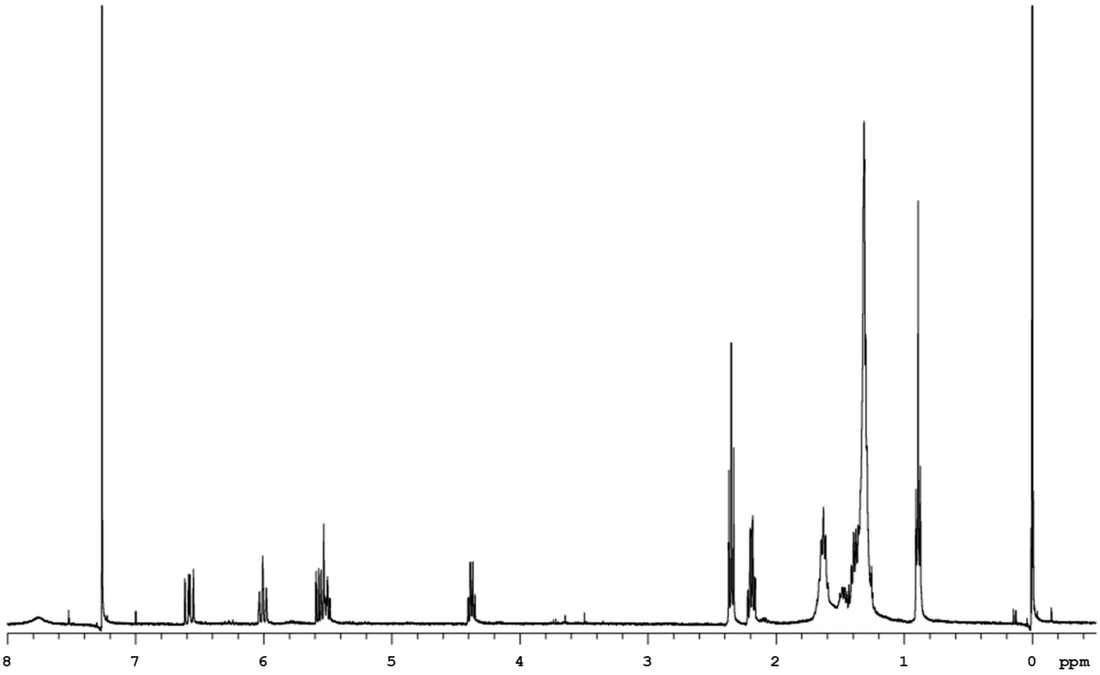

NMR chemical shifts of the C9-C14 portion of 9-10*E*,12*Z*-LAOOH

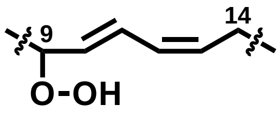

Position	ppm
9	4.38 (q, *J* = 6.8, 8.0 Hz)
10	5.48-5.59 (m)
11	6.58 (dd, *J* = 10.8, 15.2 Hz)
12	6.01 (q, *J* = 10.8 Hz)
13	5.48-5.59 (m)
14	2.19 (dq, *J* = 1.2, 7.6 Hz)

Supplementary Information 2

B

^{1}H NMR (400 MHz, $CDCl_3$)

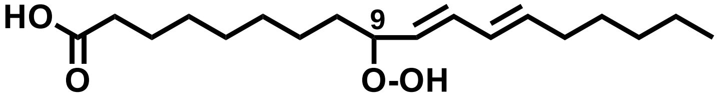

9-10*E*,12*E*-LAOOH

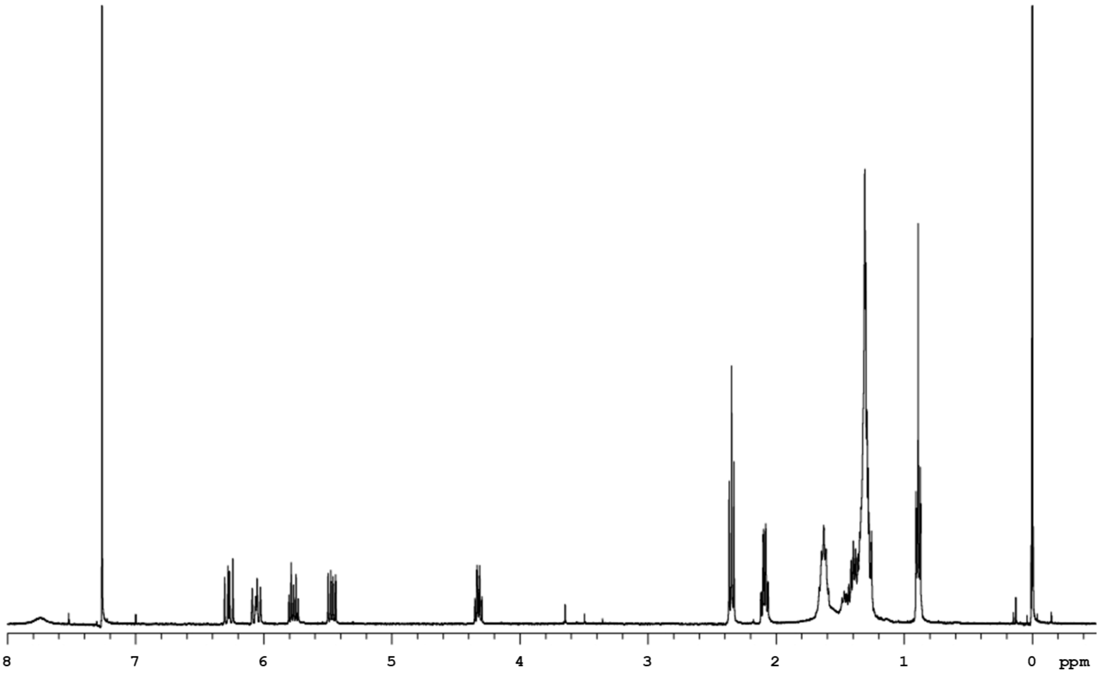

NMR chemical shifts of the C9-C14 portion of 9-10*E*,12*E*-LAOOH

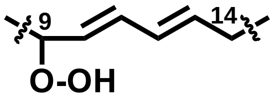

Position	ppm
9	4.32 (q, *J* = 6.8, 8.0 Hz)
10	5.47 (dd, *J* = 8.4, 15.2 Hz)
11 or 12	6.06 (dd, *J* = 10.4, 15.2 Hz)
	6.27 (dd, *J* = 10.4, 15.2 Hz)
13	5.77 (quin, *J* = 6.8, 15.2 Hz)
14	2.09 (q, *J* = 6.8 Hz)

Supplementary Information 2

C

^{1}H NMR (400 MHz, $CDCl_3$)

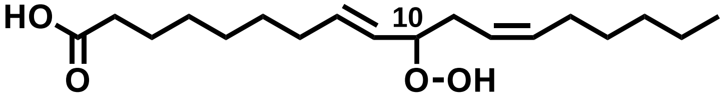

10-8*E*,12*Z*-LAOOH

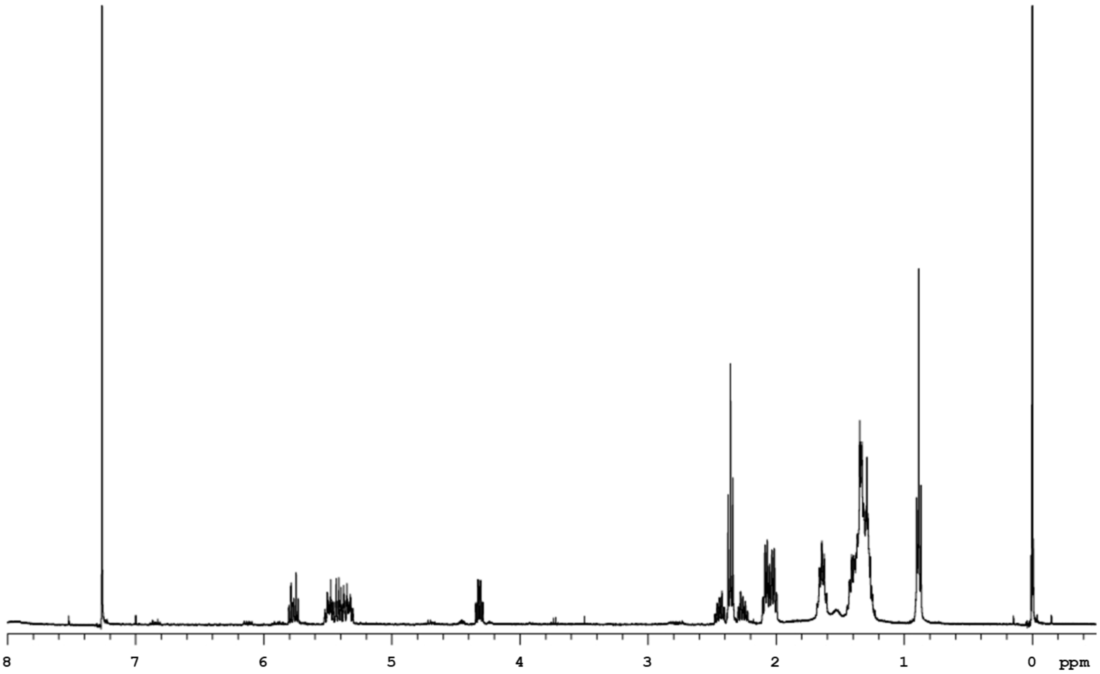

NMR chemical shifts of the C7-C14 portion of 10-8*E*,12*Z*-LAOOH

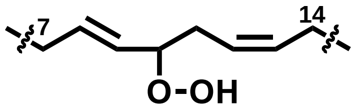

Position	ppm
7	2.00-2.10 (m)
8	5.77 (dt, *J* = 6.8, 15.6 Hz)
9	5.31-5.53 (m)
10	4.32 (q, *J* = 6.4, 8.0 Hz)
11	2.22-2.47 (m)
12	5.31-5.53 (m)
13	5.31-5.53 (m)
14	2.00-2.10 (m)

Supplementary Information 2

D

^{1}H NMR (400 MHz, $CDCl_3$)

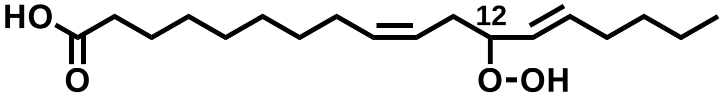

12-9*Z*,13*E*-LAOOH

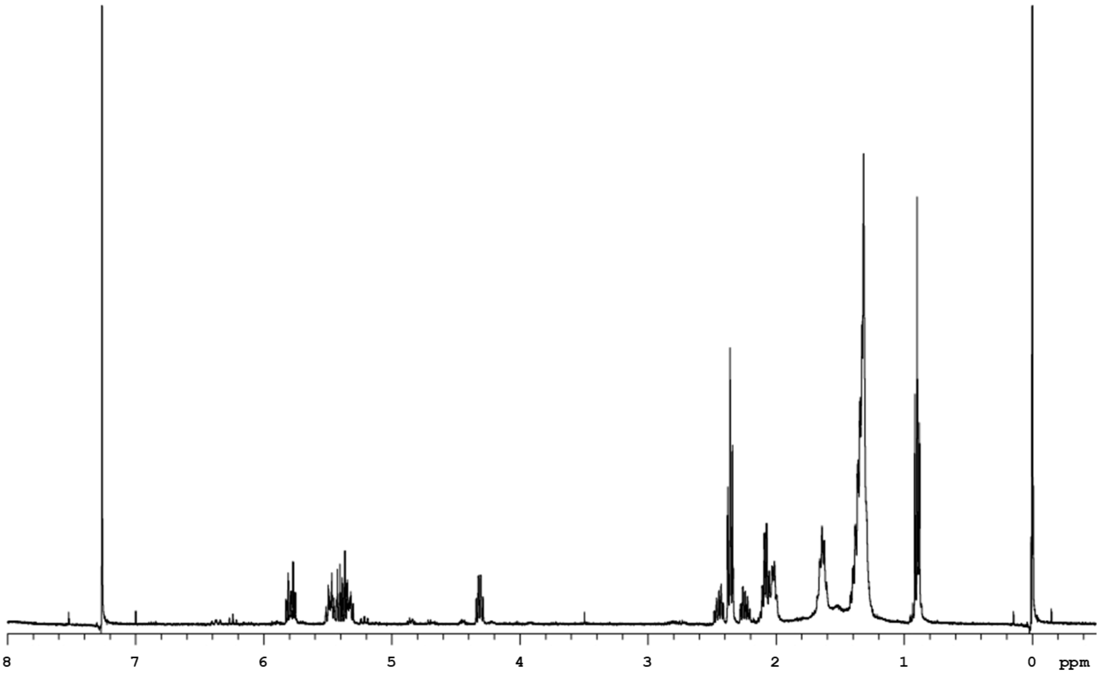

NMR chemical shifts of the C8-C15 portion of 12-9*Z*,13*E*-LAOOH

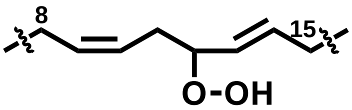

Position	ppm
8	1.99-2.14 (m)
9	5.30-5.51 (m)
10	5.30-5.51 (m)
11	2.20-2.48 (m)
12	4.31 (q, *J* = 6.4, 7.6 Hz)
13	5.30-5.51 (m)
14	5.79 (dt, *J* = 6.4, 15.2 Hz)
15	1.99-2.14 (m)

Supplementary Information 2

E

^{1}H NMR (400 MHz, $CDCl_3$)

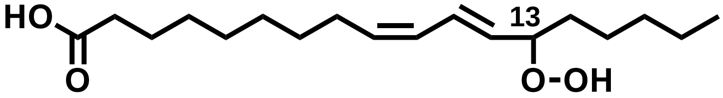

13-9*Z*,11*E*-LAOOH

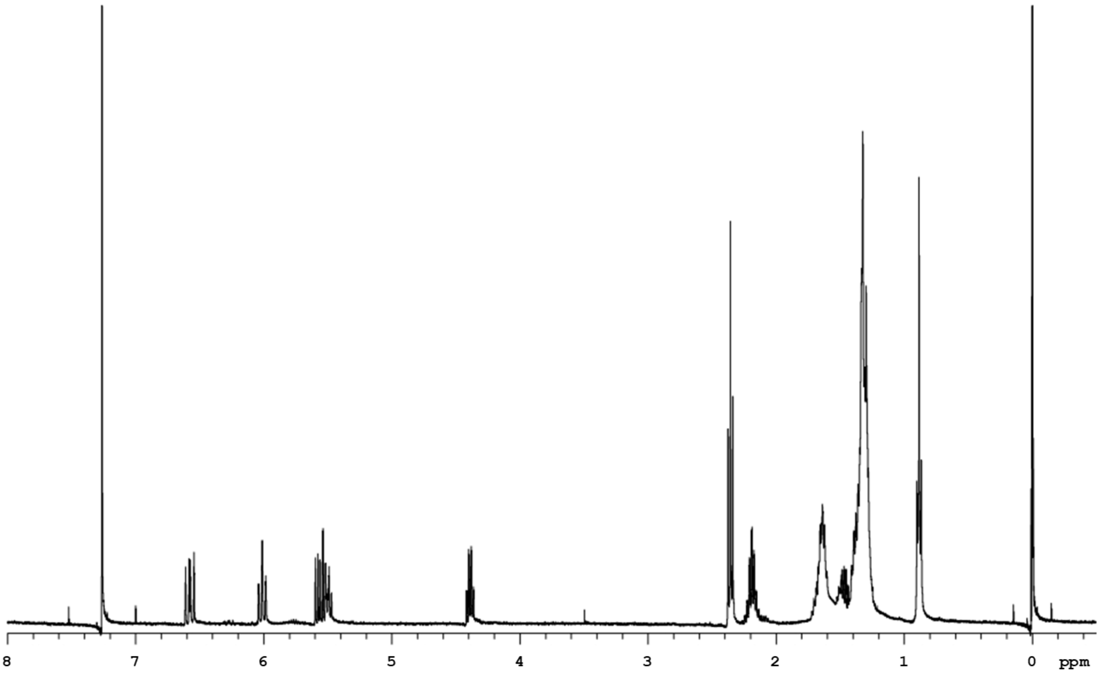

NMR chemical shifts of the C8-C13 portion of 13-9*Z*,11*E*-LAOOH

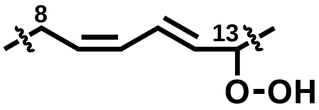

Position	ppm
8	2.19 (m)
9	5.47-5.60 (m)
10	6.01 (t, *J* = 11.2 Hz)
11	6.58 (dd, *J* = 11.2, 15.2 Hz)
12	5.47-5.60 (m)
13	4.39 (q, *J* = 6.8, 8.0 Hz)

Supplementary Information 2

F

^{1}H NMR (400 MHz, $CDCl_3$)

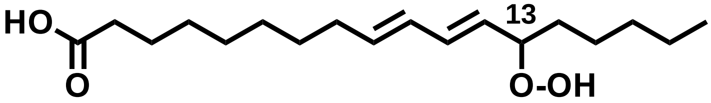

13-9*E*,11*E*-LAOOH

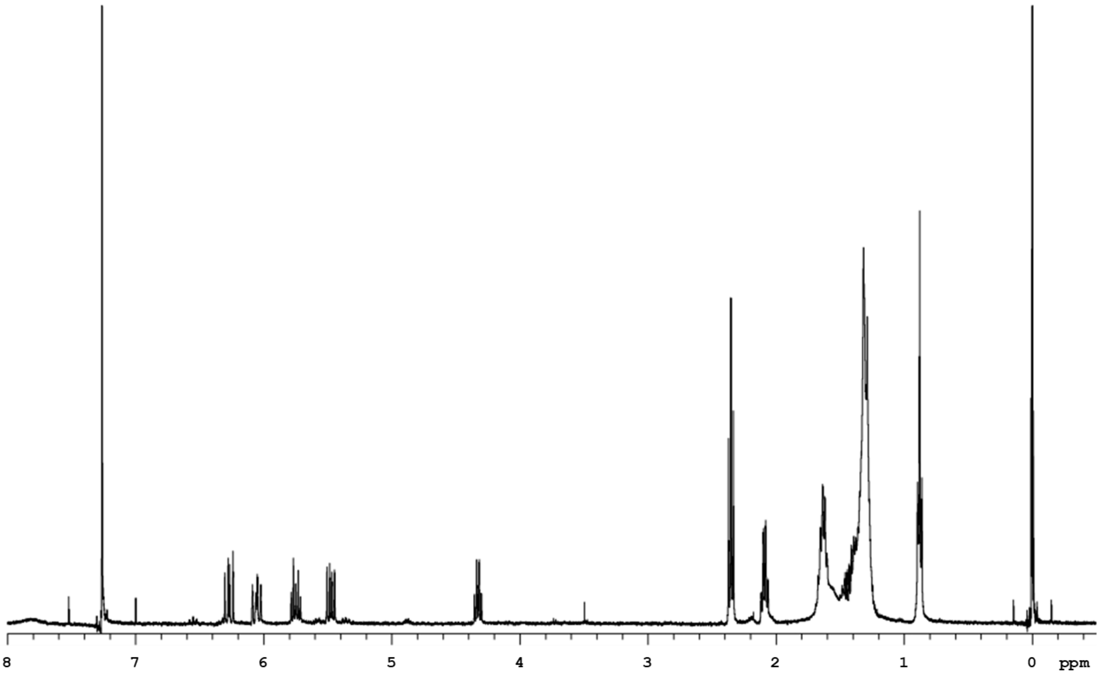

NMR chemical shifts of the C8-C13 portion of 13-9*E*,11*E*-LAOOH

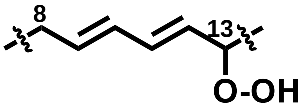

Position	ppm
8	4.33 (q, *J* = 6.4, 8.4 Hz)
9	5.75 (quin, *J* = 8.0, 15.2 Hz)
10 or 11	6.06 (dd, *J* = 11.2, 15.2 Hz)
	6.27 (dd, *J* = 10.4, 15.2 Hz)
12	5.48 (dd, *J* = 8.4, 15.2 Hz)
13	2.09 (q, *J* = 6.8 Hz)

Supplementary Information 3

^{1}H NMR spectra of ELAOOH isomers (9-10*E*,12*Z*-ELAOOH (A), 9-10*E*,12*E*-ELAOOH (B), 10-8*E*,12*Z*-ELAOOH (C), 12-9*Z*,13*E*-ELAOOH (D), 13-9*Z*,11*E*-ELAOOH (E), 13-9*E*,11*E*-ELAOOH (F)). Detailed analytical conditions are described in the Materials and Methods section.

Supplementary Information 3

A

^{1}H NMR (400 MHz, $CDCl_3$)

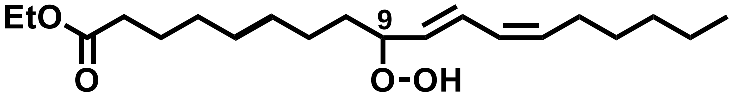

9-10*E*,12*Z*-ELAOOH

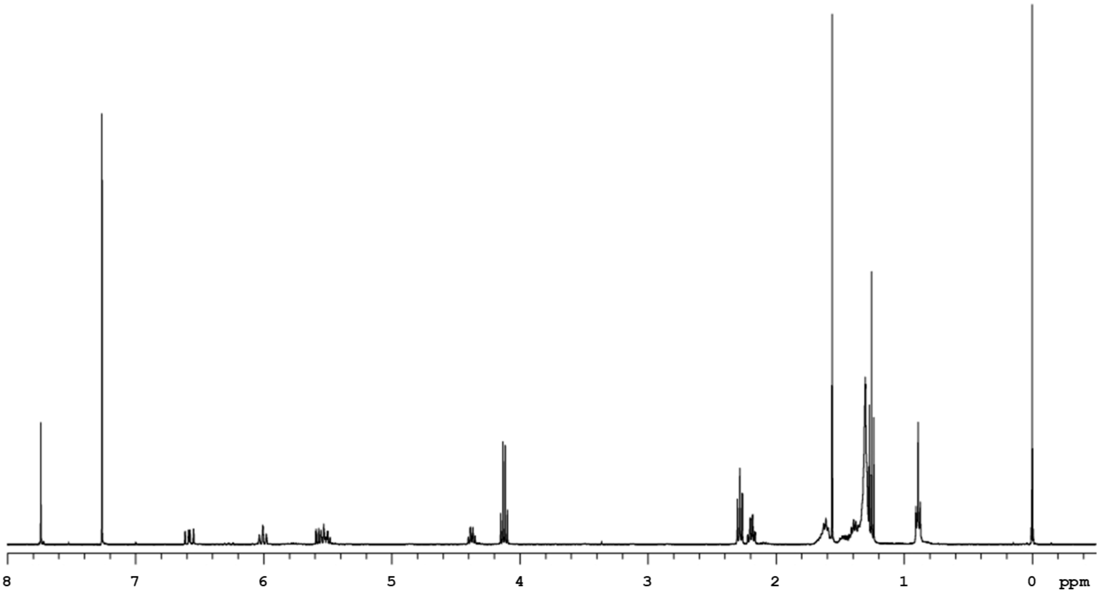

NMR chemical shifts of the C9-C14 portion of 9-10*E*,12*Z*-ELAOOH

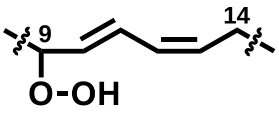

Position	ppm
9	4.38 (q, *J* = 6.8, 8.0 Hz)
10	5.48-5.59 (m)
11	6.58 (dd, *J* = 11.2, 15.2 Hz)
12	6.01 (t, *J* = 10.8, 11.2 Hz)
13	5.48-5.59 (m)
14	2.19 (dq, *J* = 1.2, 7.6 Hz)

Supplementary Information 3

B

^{1}H NMR (400 MHz, $CDCl_3$)

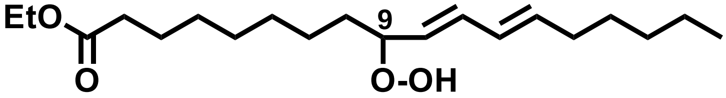

9-10*E*,12*E*-ELAOOH

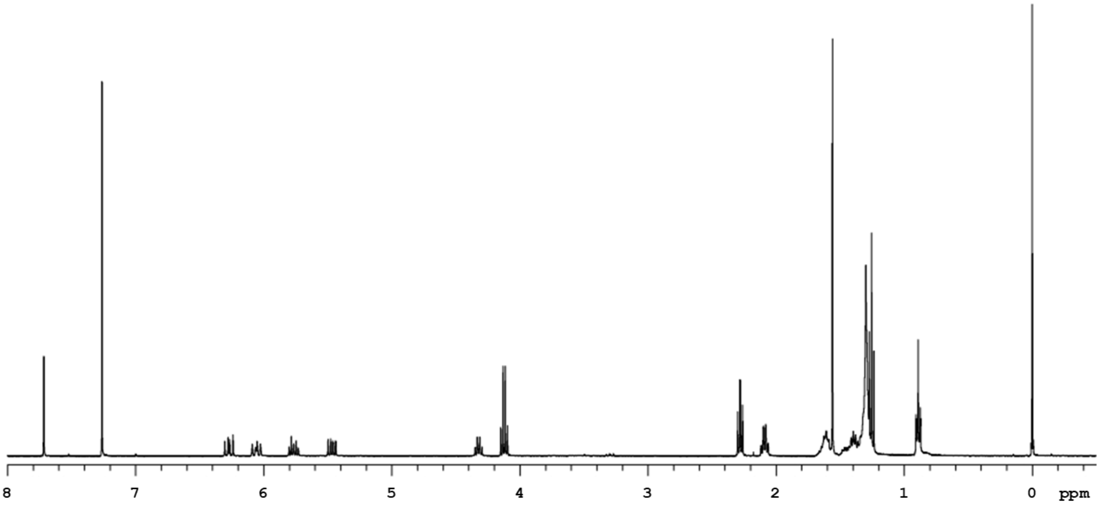

NMR chemical shifts of the C9-C14 portion of 9-10*E*,12*E*-ELAOOH

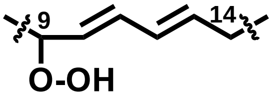

Position	ppm
9	4.32 (q, *J* = 6.8, 8.4 Hz)
10	5.47 (dd, *J* = 8.4, 15.2 Hz)
11 or 12	6.06 (dd, *J* = 10.4, 15.2 Hz)
	6.27 (dd, *J* = 10.4, 15.2 Hz)
13	5.77 (quin, *J* = 6.8, 15.2 Hz)
14	2.19 (q, *J* = 6.8 Hz)

Supplementary Information 3

C

^{1}H NMR (400 MHz, $CDCl_3$)

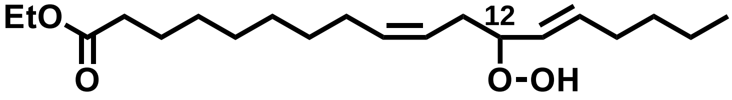

12-9*Z*,13*E*-ELAOOH

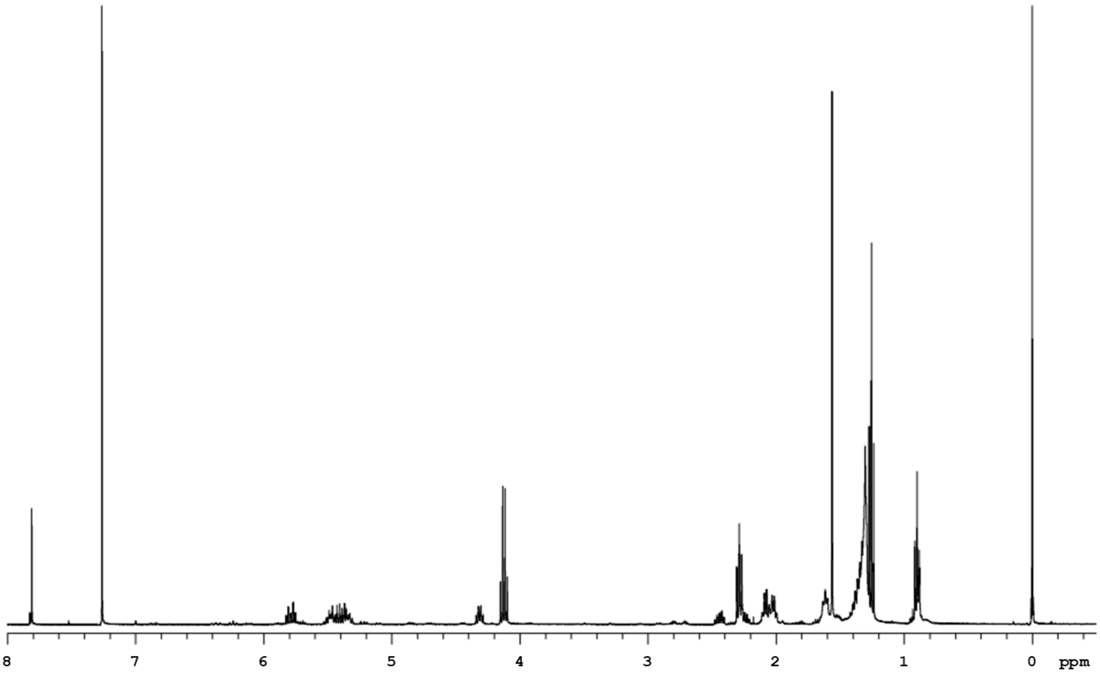

NMR chemical shifts of the C8-C15 portion of 12-9*Z*,13*E*-ELAOOH

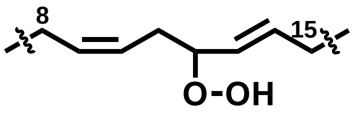

Position	ppm
8	2.00-2.11 (m)
9	5.31-5.53 (m)
10	5.31-5.53 (m)
11	2.21-2.48 (m)
12	4.31 (q, *J* = 6.8, 8.0 Hz)
13	5.31-5.53 (m)
14	5.79 (dt, *J* = 6.8, 15.2 Hz)
15	2.00-2.11 (m)

Supplementary Information 3

D

^{1}H NMR (400 MHz, $CDCl_3$)

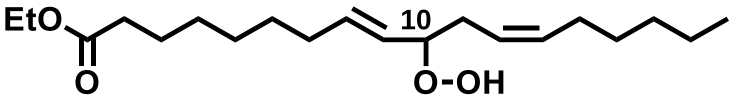

10-8*E*,12*Z*-ELAOOH

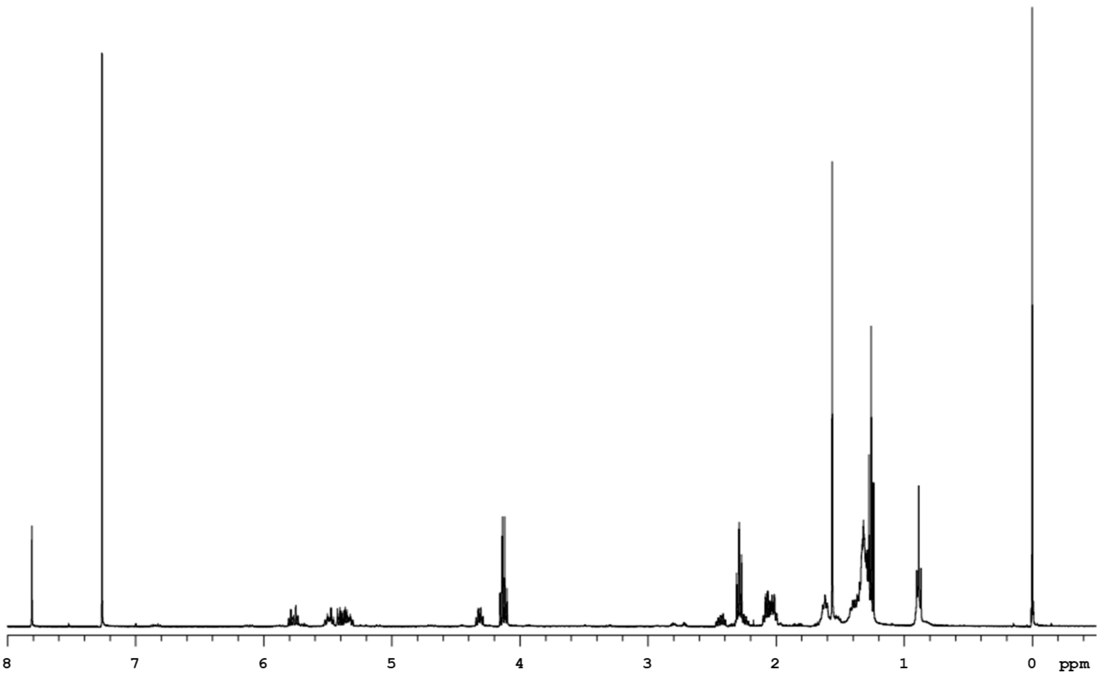

NMR chemical shifts of the C7-C14 portion of 10-8*E*,12*Z*-ELAOOH

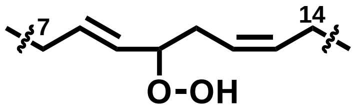

Position	ppm
7	2.00-2.10 (m)
8	5.77 (dt, *J* = 7.2, 15.6 Hz)
9	5.31-5.52 (m)
10	4.32 (q, *J* = 6.8, 8.0 Hz)
11	2.22-2.47 (m)
12	5.31-5.52 (m)
13	5.31-5.51 (m)
14	2.00-2.10 (m)

Supplementary Information 3

E

^{1}H NMR (400 MHz, $CDCl_3$)

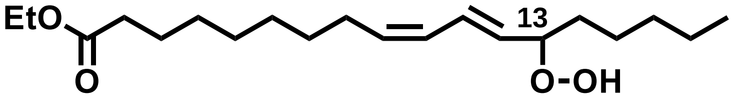

13-9*Z*,11*E*-ELAOOH

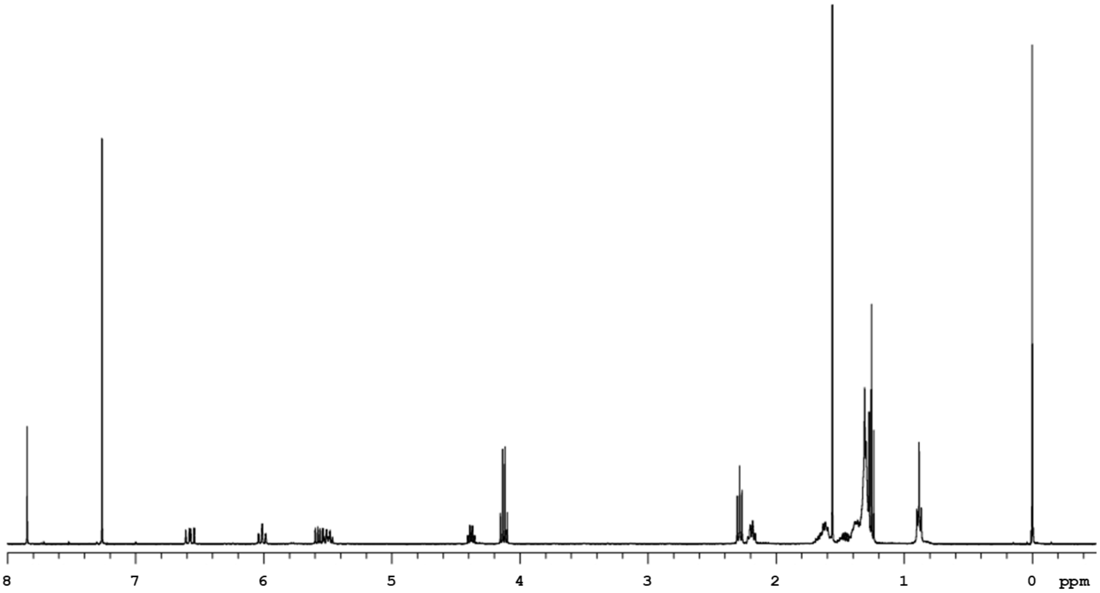

NMR chemical shifts of the C8-C13 portion of 13-9*Z*,11*E*-ELAOOH

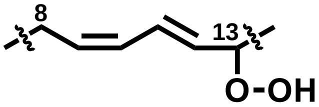

Position	ppm
8	2.19 (m)
9	5.50 (dt, *J* = 7.6, 10.4 Hz)
10	6.01 (t, *J* = 10.4, 11.2 Hz)
11	6.58 (dd, *J* = 11.2, 15.2 Hz)
12	5.57 (dd, *J* = 8.0, 15.2 Hz)
13	4.38 (dt, *J* = 6.8, 8.0 Hz)

Supplementary Information 3

F

^{1}H NMR (400 MHz, $CDCl_3$)

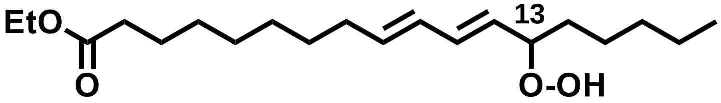

13-9*E*,11*E*-ELAOOH

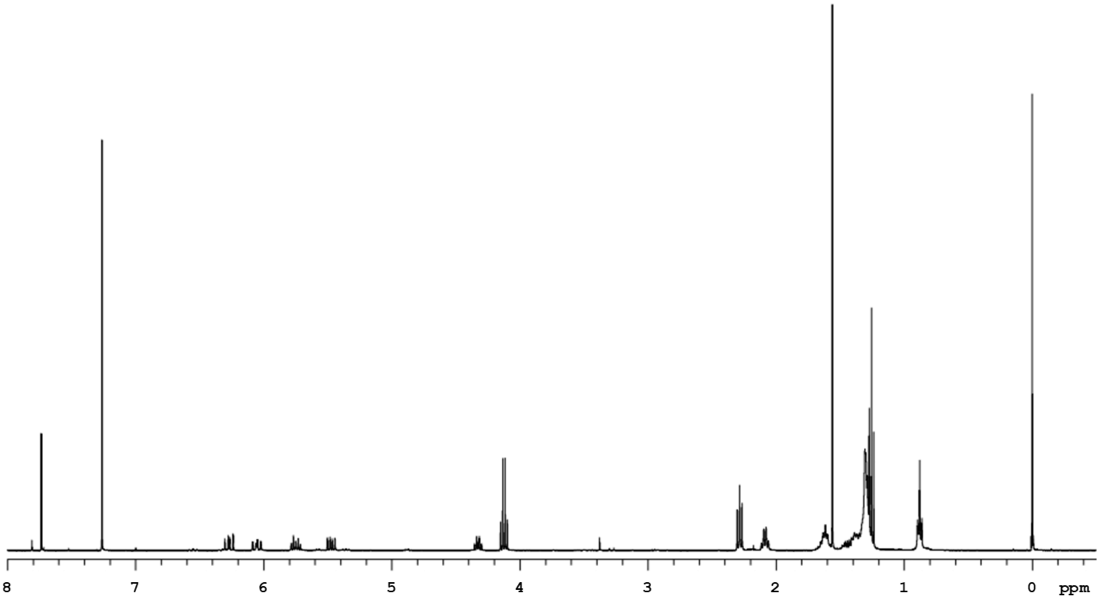

NMR chemical shifts of the C8-C13 portion of 13-9*E*,11*E*-ELAOOH

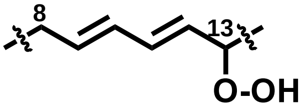

Position	ppm
8	2.09 (q, *J* = 6.8 Hz)
9	5.75 (dt, *J* = 6.8, 15.2 Hz)
10 or 11	6.06 (ddd, *J* = 1.2, 10.4, 15.2 Hz)
	6.27 (dd, *J* = 10.4, 15.2 Hz)
12	5.47 (dd, *J* = 8.0, 15.2 Hz)
13	4.33 (dt, *J* = 6.8, 8.0 Hz)
